# The school policy, social, and physical environment and change in adolescent physical activity: An exploratory analysis using the LASSO

**DOI:** 10.1371/journal.pone.0249328

**Published:** 2021-04-08

**Authors:** Campbell Foubister, Esther M. F. van Sluijs, Anna Vignoles, Paul Wilkinson, Edward C. F. Wilson, Caroline H. D. Croxson, Helen Elizabeth Brown, Kirsten Corder

**Affiliations:** 1 UKCRC Centre for Diet and Activity Research (CEDAR), MRC Epidemiology Unit, University of Cambridge, Cambridge, United Kingdom; 2 Faculty of Education, University of Cambridge, Cambridge, United Kingdom; 3 Department of Psychiatry, University of Cambridge, Cambridgeshire and Peterborough NHS Foundation Trust, Cambridge, United Kingdom; 4 Health Economics Group, Norwich Medical School, University of East Anglia, Norwich, United Kingdom; 5 Nuffield Department of Primary Care Health Sciences, University of Oxford, Oxford, United Kingdom; Edinburgh Napier University, UNITED KINGDOM

## Abstract

**Purpose:**

We examined the association between the school policy, social and physical environment and change in adolescent physical activity (PA) and explored how sex and socioeconomic status modified potential associations.

**Methods:**

Data from the GoActive study were used for these analyses. Participants were adolescents (n = 1765, mean age±SD 13.2±0.4y) from the East of England, UK. Change in longitudinal accelerometer assessed moderate-to-vigorous physical activity (MVPA) was the outcome. School policy, social and physical environment features (n = 267) were exposures. The least absolute shrinkage and selection operator variable selection method (LASSO) was used to determine exposures most relevant to the outcome. Exposures selected by the LASSO were added to a multiple linear regression model with estimates of change in min/day of MVPA per 1-unit change in each exposure reported. Post-hoc analyses, exploring associations between change in variables selected by the LASSO and change in MVPA, were undertaken to further explain findings.

**Findings:**

No school policy or physical environment features were selected by the LASSO as predictors of change in MVPA. The LASSO selected two school social environment variables (participants asking a friend to do physical activity; friend asking a participant to do physical activity) as potential predictors of change in MVPA but no significant associations were found in subsequent linear regression models for all participants (β [95%CI] -1.01 [-2.73;0.71] and 0.65 [-2.17;0.87] min/day respectively). In the post-hoc analyses, for every unit increase in change in participants asking a friend to do PA and change in a friend asking participants to do PA, an increase in MVPA of 2.78 (1.55;4.02) and 1.80 (0.48;3.11) min/day was predicted respectively.

**Conclusions:**

The school social environment is associated with PA during adolescence. Further exploration of how friendships during adolescence may be leveraged to support effective PA promotion in schools is warranted.

## Introduction

Physical activity in adolescence is positively associated with improvements to metabolic health [1], mental health [2] and maths performance [3]. However, adolescents’ physical activity levels are much lower than recommended [4] and there is therefore a need to find ways to increase adolescent physical activity.

Schools are often targeted as settings for physical activity promotion but previous school-based physical activity interventions have not been effective at increasing physical activity over the whole day [5]. A common critique of school-based physical activity strategies is the focus on behaviour change at the individual level and lack of acknowledgement of the multidimensional influences on adolescent physical activity within schools [6]. Adopting a socioecological approach and intervening at multiple levels to address the wider school environment may bring rise to sustained increases in adolescent physical activity [7]. This may also support effective intervention implementation, which is a major challenge faced by schools, particularly when translating evidence-based interventions into routine practice [8].

The school policy, social and physical environment can influence adolescent physical activity and may contribute towards adolescent health inequalities [9]; yet has been understudied. School physical environment features, such as settings for specific activities (e.g. indoor gym), the school social environment, including perceived teacher support for physical activity, and the school policy environment, for example provision of opportunities to be physically active, have been shown to positively influence adolescent physical activity [9]. The school environment may have a long-term impact on physical activity during adulthood [10]. As physical activity declines during adolescence [10] it is important to identify and target factors associated with change in adolescent physical activity. However, the association between adolescent physical activity and other features of the school environment–including school funding levels, peer support for physical activity and the overall school ethos surrounding physical activity is not clear. Existing evidence is largely comprised of cross-sectional investigations from the United States that use self-report physical activity measures [9]. Few studies in the United Kingdom have explored which elements of the school policy, social and physical environment influence adolescent physical activity and for whom this may be more or less likely.

Inequalities may arise within schools despite apparent exposure to the same school environment as the most socioeconomically disadvantaged adolescents have been found to experience school differently to those from more affluent backgrounds and have lower levels of self-reported physical activity than more affluent adolescents [11]. Further exploration of the association between the school environment and adolescent physical activity and potential moderating role of socioeconomic status (SES) using device-measured physical activity would allow stronger conclusions to be drawn on how inequalities may widen within schools. Similarly, findings from the most recent systematic review in this area–that collated both quantitative and qualitative evidence–found features of the school environment (such as access to equipment, fostering of autonomy, competence and relatedness and provision of extracurricular opportunities) impacted boys and girls differently [9]. Further high-quality research is required to establish whether certain school environment features are mechanisms underlying inequalities in physical activity during adolescence.

A large number of school environment features plausibly relate to adolescent physical activity. The primary objective of this study is to explore associations between the school policy, social and physical environment and change in adolescent physical activity to test many potential predictors of change as a hypotheses-generating exercise. The secondary objective of this study is to ascertain how sex and SES modify the relationship between the school policy, social and physical environment and change in adolescent physical activity.

## Methods

### Study sample

This paper describes secondary analyses of the GoActive study data [12], a randomised controlled trial to increase moderate-to-vigorous physical activity among adolescents. The results of the GoActive trial showed that adolescents become more physically inactive over time with no difference found between control and intervention participants’ physical activity [13], therefore the cohort for this study included all participants from control and intervention schools. Methods of the GoActive trial have been described in detail elsewhere [12]; a brief summary of the methods relevant to the current analysis is provided below.

### Recruitment and ethics

Baseline data collection took place September 2016-January 2017 with follow up April-July 2018. Participants (all year 9 students, 13-14years) were recruited from 16 non-fee-paying, co-educational secondary schools across Cambridgeshire and Essex, United Kingdom. Ethical approval for the GoActive study was obtained from the Cambridge Psychology Research Ethics committee (PRE.126.2016). The GoActive study was prospectively registered (ISRCTN31583496).

### Outcome measure

MVPA was measured by wrist-worn accelerometers (Axivity AX3, United Kingdom). Participants were asked to wear the accelerometers for 7 days continuously on their non-dominant wrist. Trained research assistants fitted the devices during data collection visits in schools and participants were encouraged to return the accelerometer to their tutor group (homeroom) teachers at the end of the 7 days. Participants were also given a freepost envelope to return accelerometers to the study team. A novel data processing approach was developed [13] in which data were processed by first separating days of possible wear into quadrants: morning (6am-12pm), afternoon (12pm-6pm), evening (6pm-midnight), and night (midnight-6am). Participants were included if over six hours of wear time spread over at least two days was recorded in each of the first three quadrants (i.e. ≥6 hours from 7 possible mornings, ≥6 hours from 7 possible afternoons, and ≥6 hours from 7 possible evenings). The night quadrant was considered sleep time. An individual hour was included for analyses when at least 70% of possible wear time was recorded. A diurnal adjustment was used to reduce bias arising from the 24-hour wear protocol. Accelerometer output was processed to provide average daily minutes of MVPA at baseline and follow up which is equivalent to ≥2000 ActiGraph counts per minute [12]. Mean wear time was … Change in average daily minutes of MVPA was then calculated via the following equation:

Change in average daily minutes of MVPA = Follow up MVPA–Baseline MVPA

Change in average daily minutes of MVPA was selected as the outcome measure–as opposed to change in school time MVPA–because certain school environment features have a theoretical basis to influence physical activity both during and outside of school time. For example, schools may allow access to school sports facilities during weekdays after school or support extracurricular activities at weekends [14]

### Exposures

A full list of exposure variables can be found in S1 Table. School level policy, social and physical environment features (n = 98)–including break time length, physical activity opportunities at school and hours of PE per week–were self-reported by contact teachers at all schools in a survey based on a questionnaire previously used in schools in the East of England as part of the Speedy-3 Study [15]. The survey used to collect these features can be found in S1 File. Individual level (n = 3) friendship support for physical activity was derived using three items from the European Youth Heart Study [16] self-reported by participants in baseline questionnaires. Additional school level exposure variables (n = 167)–including Office for Standards in Education (Ofsted) rating and school funding levels) were collected from publicly available data based on previous evidence [9] and after discussion with school-based physical activity promotion stakeholders from policy and practice (GoActive Final Report, 2020). School funding levels and Ofsted overall effectiveness rating–which assesses schools on factors including motivational climate, school connectedness, teacher support of pupils, teacher leadership behaviours and teacher and pupil skill building [17] may provide insight into the challenging-to-measure school social environment. These data were obtained from the following website: https://schools-financial-benchmarking.service.gov.uk/.

### Descriptive data and covariates

Sociodemographic data (age, sex, ethnicity, language spoken at home, religious affiliation, family structure, family SES and BMI) were self-reported by participants at baseline. Participants reported their ethnicity from 20 response options and values were recoded to the following categories: White; Mixed ethnicity; Asian; Black; Other ethnicity. Religious affiliation was reported from eight response options and values were recoded to the following categories: No religion; Any religion. Language spoken at home was reported from 26 response options and values were recoded to the following categories: English only; English and other language(s); Other language(s) not English). Participants reported one or two main care-givers from eight response options and family structure was recoded to the following categories: Birth Mother and Father; Any other family structure. Participants reported six items from the Family Affluence Scale [18] (family car ownership, holidays, computers, availability of bathrooms, dishwasher ownership and having their own bedroom) and values were summed (possible range 0–13) and used as a proxy measure of family SES (affluence: low = 0–6, medium = 7–9, high = 10–13) [19]. Height and weight were recorded by research staff trained in anthropometric assessment and BMI z-score was calculated from height, weight, age, and sex [20].

### Statistical analysis

As a large number of school environment features plausibly relate to physical activity, the least absolute shrinkage and selection operator (LASSO) variable selection method was used to determine the features of the school environment that are most relevant to change in MVPA [21]. The tuning penalty applied to the LASSO–which determines the amount of shrinkage applied by capping the sum of the absolute coefficients—was the Extended Bayesian information criterion which accounts for collinearity [22]. School environment features selected by the LASSO were added to a multiple linear regression model to identify features significantly associated with change in physical activity. Change in MVPA from 13y to 15y (average follow up = 18 months) acted as the dependent variable and the school policy, social and physical environment features served as independent variables. Estimates of change in minutes per day of MVPA per 1-unit change in each independent variable were reported. As GoActive was a cluster-randomized controlled trial and this study used data from the whole cohort, treatment group (intervention and control) was added to each LASSO, along with adjustments for baseline physical activity, baseline BMI and sex. This process was repeated separately by sex (Male, and Female) and SES (Low, Medium, and High) to stratify the analyses, looking for differences in effect. To account for the non-independence of participants (clustering within schools and SES), robust standard errors were calculated. LASSO variable selection has previously been used to identify the most meaningful correlates of adolescent physical activity using self-report physical activity data [23]. The LASSO method is different from traditional approaches to variable selection such as the stepwise approach which uses p-values to determine which variables to include. Instead, the LASSO shrinks the absolute value of the magnitude of regression coefficients of unrelated school environment features to zero by regularization. The main driver for using this method was that a large number of school environment features plausibly related to change in MVPA. As this was an exploratory analysis, intended to test many potential predictors of change as a hypothesis generating exercise, LASSO variable selection was used to allow only the most relevant school environment variables to physical activity to be included in the model. This was in order to produce a robust and parsimonious model via minimizing prediction error and improving interpretability [24]. The number of independent variables that regression analysis is capable of dealing with is a subject of contention [25]. The LASSO approach is justified to counteract the rise in biases (e.g., overfitting, multicollinearity) when there are greater than five independent variables in a multiple regression equation [26] To improve our understanding of these findings, post hoc analyses were performed to explore the association between change in the variables selected by the LASSO and change in MVPA. Statistical analyses was performed in STATA version 15.1 [27, 28] (using the lassopack (v1.2) installation package to run the LASSO.

## Results

1765 participants (51% female) had both baseline and follow up physical activity data from which to derive change in MVPA. Participant characteristics are displayed in Table 1. Participants were predominantly of White British ethnicity, medium or high SES, had no religious affiliation, spoke English only at home, and lived in a traditional family structure.

**Table 1:** Baseline descriptive characteristics.

	Male	Female
	n = 879	n = 906
**Age (mean (SD) in years)**	13.23 (0.42)	13.21 (0.41)
**BMI SDS (mean (SD)**	0.23 (1.19)	0.36 (1.20)
**Ethnicity (N (%))**		
White	740 (85.0)	789 (87.9)
Mixed/multiple ethnic background	66 (7.6)	39 (4.3)
Asian or Asian British	39 (4.5)	33 (3.7)
Black or Black British	17 (2.0)	21 (2.3)
Other Ethnic Group	9 (1.0)	16 (1.8)
**Family SES (N (%))**		
Low	89 (10.2)	114 (12.7)
Medium	345 (39.5)	401 (44.6)
High	439 (50.3)	385 (42.8)
**Religious affiliation (N (%))**		
No religion	662 (77.2)	674 (75.9)
Any religion	196 (22.8)	214 (24.1)
**Language spoken at home (N (%))**		
English only	795 (93.2)	824 (93.3)
English and other language(s)	16 (1.9)	21 (2.4)
Other language(s) not English	42 (4.9)	38 (4.3)
**Family structure (N (%))**		
Mother and Father	742 (85.2)	716 (79.6)
Any other family structure	129 (14.8)	184 (20.4)

### Main analyses

A total of 267 school environment features were included in the LASSO variable selection technique, a full list of variables included in the LASSO is shown in S1 Table. Estimates of effects for the variables selected by the LASSO and included in the main analyses are shown in Table 2. No school policy or physical environment features were selected by the LASSO as potential predictors of change in MVPA. Only 3 variables (baseline MVPA, having a friend ask participants to do physical activities or play sports with them, and a participant asking a friend to do physical activities or play sports with them) were selected and added to the regression model when running the analyses with all participants. In this regression analysis, only baseline MVPA was found to be independently negatively associated with change in MVPA.

**Table 2:** Estimated effects of the variables selected by the LASSO on change in MVPA during adolescence.

Variable	All Participants	Boys	Girls	Low SES	Medium SES	High SES
Baseline MVPA	**-0.54 (-0.65, -0.45)**	**-0.59 (-0.71, -0.47)**	**-0.47 (-0.64, -0.30)**	**-**	**-0.48 (-0.64, -0.32)**	**-0.58 (-0.72, -0.44)**
Participant asks friends to do physical activity	-1.01 (-2.73, 0.71)	-0.63 (-3.57, 2.31)	-	-	**-2.57 (-3.71, -1.43)**	-1.76 (-3.61, 0.08)
Friend asks participant to do physical activity	0.65 (-2.17, 0.87)	**-2.40 (-4.78, -0.02)**	-	-	-	-

Estimates (β (95%CI)) are from linear regression models adjusted for other variables in the model. Estimates can be interpreted as change in average daily minutes of MVPA per 1-unit change in the independent variable. Bold denotes significant association.

#### Stratified analyses by sex

For boys (n = 879), the LASSO selected the same 3 variables but only baseline MVPA and boys having a friend ask them to do physical activities or play sports with them were found to be associated with change in MVPA in the regression model. For every unit increase in number of times per week male adolescents have a friend ask them to do physical activities or play sports with them, a decrease in physical activity of (β (95%CI)) -2.40 (-4.78, -0.02) minutes of MVPA is predicted, as shown in Table 2. When repeating the analysis process for girls only, no school policy, social or physical environment features were selected by the LASSO and explored in regression analyses.

#### Stratified analyses by SES

For adolescents with a low SES, no school policy, social or physical environment variables were selected by the LASSO. For adolescents with a medium SES, the LASSO selected baseline MVPA and participants asking friends to do physical activities or play sports with them to be included in regression models. For every unit increase in number of times per week participants with a medium SES asked their friends to do physical activity or play sports with them, a change in physical activity of -2.57 (-3.71, -1.43) minutes of MVPA is predicted. For adolescents with a high SES, the LASSO selected having a friend ask them to do physical activities or play sports with them but only baseline MVPA was found to be significantly associated in regression models.

### Post hoc analyses

Longitudinal associations between change in friendship support for physical activity and change in MVPA are shown in Table 3. For all participants, both friendship support for physical activity variables were positively associated with change in MVPA. For every unit increase in change in participants asking friends to do physical activity, a change in MVPA of 2.78 (1.55, 4.02) minutes per day is predicted. For every unit increase in change in friends asking participants to do physical activity, a change in MVPA of 1.80 (0.48, 3.11) minutes is predicted.

**Table 3:** Estimate effects of change in the variables selected by the LASSO and change in MVPA.

Variable	All Participants	Boys	Girls	Low SES	Medium SES	High SES
Change in participant asking friend to do physical activity	**2.78 (1.55, 4.02)**	**3.70 (2.12, 5.27)**	1.22 (-0.47, 2.92)	2.73 (-1.57, 7.03)	**3.18 (1.43, 4.92)**	**2.36 (0.24, 4.48)**
Change in friend asking participant to do physical activity	**1.80 (0.48, 3.11)**	**2.65 (0.84, 4.45)**	0.25 (-1.49, 1.99)	3.42 (-0.13, 6.97)	1.47 (-0.23, 3.17)	1.75 (-0.19, 3.69)

Estimates (β (95%CI)) are from linear regression models adjusted for baseline variable values. Estimates can be interpreted as change in average daily minutes of MVPA per 1-unit change in the independent variable. Bold denotes significant association.

In sub-group analyses by sex, for boys only, positive associations were found for both measures of friendship support for physical activity; whereas, for girls only, no associations were found for either measure of friendship support for physical activity. In sub-group analyses by SES, positive associations were found for participants with a medium and High SES, but no associations were found for participants with a low SES.

## Discussion

The primary objective of this study was to explore associations between the school policy, social and physical environment and change in adolescent MVPA. Of a possible 267 school environment features investigated, no school policy or physical environment features were selected as potential predictors of change in MVPA during adolescence and therefore none were explored via regression analyses in our main analyses. Potential explanations for the lack of associations are unenforced school policies, subjective physical environment features, and a disconnect between what the scientific community perceive to plausibly relate to physical activity and subsequently measure vs. what school environment features actually relate to adolescent physical activity. For example, scheduling of PE throughout the school day, presence of a PE uniform policy [29] or other, largely quantitatively untested features such as school friendship networks, and relationships with teachers [9]. In our main analyses, despite two social environment features being identified by the LASSO as potential predictors of change in MVPA, neither variable was significantly associated in a priori regression analyses. Significant associations were found in a priori analyses for boys (but not girls) and for participants with a medium SES (but not participants with a low or high SES) when exploring the influence of the school social environment and change in MVPA during adolescence. In post-hoc analyses, longitudinal findings revealed positive associations between change in the social environment and change in MVPA, in line with previous research highlighting the potential for harnessing the social environment to promote physical activity during adolescence [30, 31]. However, in sub-group analyses, only boys (not girls), and only adolescents with a medium and high SES (not low SES) had positive associations.

### Main analyses findings

#### The school physical environment

No school physical environment features were identified as potential predictors of change in MVPA by the LASSO. In the most recent systematic review exploring the school environment and adolescent physical activity [9], 17 studies were found that included a total of 8 unique exposures corresponding to the school physical environment. The only consistently positively associated physical environment feature found by Morton and colleagues was activity setting (e.g. indoor gym, sports hall), whereas access to physical activity or sports equipment was consistently not associated with physical activity. In our study, none of the 33 physical environment features explored (including activity setting and size, and access to physical activity or sports equipment) were associated with change in MVPA during adolescence. It may be possible that no associations were found because of the subjective measurement of school physical environment features which could have brought rise to self-reporting biases. However, no associations between the school physical environment and adolescent physical activity have been found in one study [27] while only weekday physical activity was found in another study [28] which both used geographic information systems (GIS) as the quantification method (the current gold standard). GIS-mapped neighbourhood walkability is associated with device-measured physical activity of adolescents [32]. As adolescents accumulate physical activity across many settings (e.g. the home, the neighbourhood, and during commuting), it is plausible that other physical environments may be stronger predictors of change in MVPA during adolescence. Furthermore, the relationship between the physical environment and adolescent physical activity can be moderated by the social environment [33]. A school with a physical environment that can plausibly support physical activity (e.g. excellent equipment and activity settings) but lacking of a social or policy environment that is supportive of physical activity may limit the potential beneficial impact of the physical environment on physical activity during adolescence [9].

#### The school policy environment

This UK study found no associations between the school policy environment measures (e.g. number of hours of PE provided per week, access to sports facilities and equipment at breaks and after-school) and change in adolescent MVPA. Significant and positive changes in physical activity have been found previously when school physical activity policies are implemented effectively in the USA [34]. School physical activity policies can influence school practices by providing additional opportunities to be active throughout the school day [35]; however, schools face considerable challenges implementing physical activity policies. A lack of equipment, time, staff, and facilities can impede the implementation of school physical activity policies [36]. For these reasons, actual school practice surrounding physical activity may differ from official school physical activity policies [37]. We did not have data on the fidelity of school policies but it is plausible that school policies (e.g. formal written physical activity policies) were tokenistic and not enacted. It may be worth exploring in schools in the UK whether improving the implementation of school physical activity policies has a positive impact on physical activity during adolescence. In order to improve implementation of school physical activity policies it may be necessary to bring rise to a wider shift within schools to a climate that is supportive of physical activity.

#### The school social environment

No school social environment features were found to be associated with change in adolescent MVPA after being identified as potential predictors of change by the LASSO. In our main analyses, a lack of association is at odds with previous findings which suggest that social support for physical activity is beneficial for adolescents [38, 39] perhaps due to differences in levels of motivation. A systematic review of systematic reviews found compelling evidence that having a companion for physical activity was one of the most evidence-based socio-cultural determinants of physical activity for adolescents [40]. Social support for physical activity includes any behaviour that could facilitate another individual to be active. Types of support friends may offer each other include emotional support (e.g. encouragement and/or praise), instrumental support (e.g. providing sports equipment to play with together), informational support (e.g. feedback or advice on physical activity), co-participation (performing physical activity with each other), and modelling of behaviour [41]. Encouragement and co-participation may be particularly important for adolescents, with consistent, positive associations being found between these factors and physical activity in adolescents [31, 42]. However, it has previously been shown that encouragement from friends to be physically active may be less important for adolescents who have existing, and well-established physical activity habits [43]; hence a lack of association in this study may be explained by differences in intrinsic motivation where inactive participants feel like they require the support from their friends in order to participate in physical activity. This hypothesis assumes that adolescents who do not need to ask or be asked to do physical activity may already be intrinsically motivated to participate in physical activity which prevents a decline in physical activity for these adolescents. However, our longitudinal associations are more likely to reveal causal relationships since they account for fixed propensities to do activity that vary across individuals.

### Longitudinal posthoc findings

A positive association between change in friendship support for physical activity and change in MVPA during adolescence was found. This finding is in line with the totality of the literature surrounding friendship support for physical activity. It has consistently been demonstrated that a positive association exists between friendship support for physical activity and physical activity during adolescence [31, 41]. Our findings lend support to the hypotheses that friendship support for physical activity may be an important mechanism for continued participation in physical activity throughout adolescence [43]. However, it is not possible to rule out that effects were not attributable to reverse causation, where an increase in physical activity prompted an increase in participants asking friends or being asked by friends to do physical activity. Further research is required to help explain how friendship influences physical activity during adolescence.

Friendships during adolescence may be positively or negatively influential on physical activity [44]. It has previously been demonstrated that girls receive less friendship support for physical activity than boys [45]. Friendship support for physical activity may have a greater influence on different types of physical activity, as stronger and more consistent associations were found between friendship support for physical activity and vigorous physical activities and competitive sports which may be preferred, at the population level, by boys [42]. Adolescents often report having a higher number of same-sex friendships [46] and tend to rely upon their peers to define acceptable and desirable behaviours [47, 48]. A gendered culture within British secondary schools, where boys and girls are encouraged to participate in different physical activities; may bring rise to gendered social norms and explain differences in gender peer modelling, as previously hypothesized by Reimers, Schmidt [45]. Gender stereotyping of physical activities, where social norms consider certain physical activities to be masculine or feminine, can negatively impact physical activity participation in girls [49] as adolescents may be more likely to imitate the physical activity of their same sex peers [46] and girls are less likely to do sufficient physical activity [50]. Female adolescents may have fewer active friends (relative to boys) on which they can model desirable physical activity behaviours. Further investigation is needed to confirm these hypotheses in order to aid the development of interventions designed to overcome inequalities in physical activity between boys and girls.

An alternative explanation for the finding of a positive association for boys (and not girls), is differences in motivation for physical activity between sexes. Motivation is a significant predictor of physical activity during adolescence [51]. However, correlates and determinants of physical activity often differ by sex and variations in adolescent’s motivation to be physically active have been found between boys and girls [52]. A difference in intrinsic motivation to participate in physical activity between boys and girls may also explain why a longitudinal, positive association was found between social support and change in MVPA in boys only. For example, boys may have higher intrinsic motivation to be active than girls and might seek more opportunities to participate in physical activity with friends.

### Strengths and limitations

The main limitation of this study was the subjective quantification of school environment features which may have brought rise to self-report biases. A further limitation is that we were only able to analyse the social environment features longitudinally and it was not possible to analyse the school policy, and physical environment features longitudinally. These analyses were exploratory, rather than confirmatory. Selection/homophily or influence processes in adolescent friendship networks (e.g. whether individuals become friends and then friends influence physical activity during adolescence or whether friends select friends based on similarity in physical activity) is also a highly debated topic with mixed findings [53]. Therefore, selection bias within friendship groups may have impacted findings and identified variables should be considered potential correlates rather than causal mechanisms. It is also possible that effects were not found in participants with a low SES due to a small sample size. There is also currently no consensus in the existing literature on how to classify intensities of physical activity which brings rise to the possibility of a degree of misclassification. Strengths were the longitudinal, device-measured physical activity measurements and relatively large representative sample size. These longitudinal associations are more likely to reveal causal relationships since they account for fixed propensities to do activity that vary across individuals.

### Conclusion

This study was an exploratory analysis, intended to test many potential predictors of change in MVPA within the school policy, social, and physical environment as a hypothesis generating exercise. No policy and social environment features were selected by the LASSO as potential predictors of change in MVPA. Two social environment features were identified by the LASSO but were not associated with change in MVPA in our main analyses. Post-hoc analyses suggested friendship support for physical activity could be harnessed to increase adolescent physical activity levels. Making changes in the school environment to promote friendship support for physical activity may increase adolescent physical activity [54]. Future research to better understand the school social environment, in particular exploring how friendships during adolescence influence physical activity via social network analysis, may provide insight into how to prevent the decline in physical activity during adolescence.

## Supporting information

S1 Table Exposure variables. (DOCX)

S1 File GoActive school environment survey. (PDF)
