# Supplementary material for: The school policy, social, and physical environment and change in adolescent physical activity: An exploratory analysis using the LASSO
Source: PLoS One. 2021 Apr 8;16(4):e0249328. doi: 10.1371/journal.pone.0249328 (PMC8031174; doi:10.1371/journal.pone.0249328)
Supplement: S1 Table — (DOCX) [file pone.0249328.s001.docx]

S1 Table.

| **Variable Description** | **Response Categories** | **How data treated** |
| --- | --- | --- |
| Job role of respondent | Headteacher = 1; Deputy Headteacher =2; PE Lead = 3; Year 9 Lead = 4; Other = 5 | Categorical |
| Start time of school day | Free text e.g. 8am = 0800 | Continuous |
| End time of school day | Free text e.g. 3.20pm = 1520 | Continuous |
| Start time of morning break | Free text e.g. 11am = 1100 | Continuous |
| Duration of morning break (minutes) | Free text e.g. 15 minutes = 15 | Continuous |
| Start time of lunch break | Free text e.g. 12.15pm = 1215 | Continuous |
| Duration of lunch break (minutes) | Free text e.g. 30 minutes = 30 | Continuous |
| Have any events occurred during the measurement period that may have influenced the level of physical activity of Year 9 students (e.g. sports day)? | No = 0; Yes =1 | Dichotomous |
| Presence of planted beds containing flowers/shrubs/small trees | None = 0; Some = 1; A lot =2 | Categorical |
| Presence of trees for shade | None = 0; Some = 1; A lot =2 | Categorical |
| Presence of loud ambient noise (e.g. traffic, trains, industry) | None = 0; Some = 1; A lot =2 | Categorical |
| Presence of litter | None = 0; Some = 1; A lot =2 | Categorical |
| Presence of murals/outdoor art | None = 0; Some = 1; A lot =2 | Categorical |
| Presence of graffiti | None = 0; Some = 1; A lot =2 | Categorical |
| The grounds are shielded from the surrounding area by hedges/trees/fences | Strongly disagree = 1; Disagree = 2; Neither agree nor disagree = 3; Agree = 4; Strongly agree = 5 | Categorical |
| The grounds are generally well maintained | Strongly disagree = 1; Disagree = 2; Neither agree nor disagree = 3; Agree = 4; Strongly agree = 5 | Categorical |
| The grounds are generally free of vandalism | Strongly disagree = 1; Disagree = 2; Neither agree nor disagree = 3; Agree = 4; Strongly agree = 5 | Categorical |
| Number of pupils in Year 9 | Free text e.g. 240 pupils in year 9 = 240 | Continuous |
| Number of pupils in the whole school | Free text e.g. 1300 pupils in the whole school = 1300 | Continuous |
| Number of boys in Year 9 | Free text e.g. 124 boys in Year 9 = 124 | Continuous |
| Number of boys in the whole school | Free text e.g. 700 boys in the whole school = 700 | Continuous |
| Number of girls in Year 9 | Free text e.g. 116 girls in Year 9 = 116 | Continuous |
| Number of girls in the whole school | Free text e.g. 600 girls in the whole school = 600 | Continuous |
| Percentage of students in Year 9 for whom receive Pupil Premium | Free text e.g. 16.5% of students in Year 9 receive Pupil Premium = 16.5 | Continuous |
| Percentage of students in whole school for whom receive Pupil Premium | Free text e.g. 17% of students in whole school receive pupil premium = 17 | Continuous |
| Does your school have access to a specific indoor hall for gym or sports? | No = 0; Yes, high quality = 3; Yes, medium quality = 2; Yes, low quality = 3 | Categorical |
| Does your school have access to a shared indoor facility used for sports activities? | No = 0; Yes, high quality = 3; Yes, medium quality = 2; Yes, low quality = 3 | Categorical |
| Does your school have access to a sports or football field/pitch on school grounds? | No = 0; Yes, high quality = 3; Yes, medium quality = 2; Yes, low quality = 3 | Categorical |
| Does your school have access to an athletics track (grass or hard surface)? | No = 0; Yes, high quality = 3; Yes, medium quality = 2; Yes, low quality = 3 | Categorical |
| Does your school have access to courts (e.g. tennis, basketball including half court, netball, multicourt area)? | No = 0; Yes, high quality = 3; Yes, medium quality = 2; Yes, low quality = 3 | Categorical |
| Does your school have access to a recreational area on school grounds? | No = 0; Yes, high quality = 3; Yes, medium quality = 2; Yes, low quality = 3 | Categorical |
| Does your school have access to a wildlife garden? | No = 0; Yes, high quality = 3; Yes, medium quality = 2; Yes, low quality = 3 | Categorical |
| Does your school have access to bright or fluorescent markings on play surfaces (e.g. hopscotch, animals) | No = 0; Yes, high quality = 3; Yes, medium quality = 2; Yes, low quality = 3 | Categorical |
| Does your school have access to playground equipment (e.g. swings, slide)? | No = 0; Yes, high quality = 3; Yes, medium quality = 2; Yes, low quality = 3 | Categorical |
| Does your school have access to benches? | No = 0; Yes, high quality = 3; Yes, medium quality = 2; Yes, low quality = 3 | Categorical |
| Does your school have access to picnic tables? | No = 0; Yes, high quality = 3; Yes, medium quality = 2; Yes, low quality = 3 | Categorical |
| Does your school have access to drinking fountains? | No = 0; Yes, high quality = 3; Yes, medium quality = 2; Yes, low quality = 3 | Categorical |
| Does your school have access to uncovered cycle parking? | No = 0; Yes, high quality = 3; Yes, medium quality = 2; Yes, low quality = 3 | Categorical |
| Does your school have access to covered cycle parking? | No = 0; Yes, high quality = 3; Yes, medium quality = 2; Yes, low quality = 3 | Categorical |
| Does your school have access to an assault course? | No = 0; Yes, high quality = 3; Yes, medium quality = 2; Yes, low quality = 3 | Categorical |
| Does your school have access to a formal garden or quiet space? | No = 0; Yes, high quality = 3; Yes, medium quality = 2; Yes, low quality = 3 | Categorical |
| Does your school have access to an outdoor teaching space? | No = 0; Yes, high quality = 3; Yes, medium quality = 2; Yes, low quality = 3 | Categorical |
| Does your school have access to a vegetable or fruit garden? | No = 0; Yes, high quality = 3; Yes, medium quality = 2; Yes, low quality = 3 | Categorical |
| Does your school have access to playing fields or a local park off school grounds, which you can use? | No = 0; Yes, high quality = 3; Yes, medium quality = 2; Yes, low quality = 3 | Categorical |
| Does your school have access to purpose-built changing facilities? | No = 0; Yes, high quality = 3; Yes, medium quality = 2; Yes, low quality = 3 | Categorical |
| Does your school have access to sports equipment (e.g. gymnastics equipment)? | No = 0; Yes, high quality = 3; Yes, medium quality = 2; Yes, low quality = 3 | Categorical |
| Are the school grounds generally suitable for sport (organised or not)? | Very = 3; Somewhat = 2; Not at all = 1 | Categorical |
| Are the school grounds generally suitable for informal games (kickabout, Frisbee etc)? | Very = 3; Somewhat = 2; Not at all = 1 | Categorical |
| Are the school grounds generally suitable for general play? | Very = 3; Somewhat = 2; Not at all = 1 | Categorical |
| How many hours of physical education do pupils in Year 9 usually have per week? | Free text e.g. 2 hours of PE per week = 2 | Continuous |
| Does your school or any other organisation provide any extracurricular physical activity or sports programmes available to Year 9 before school? | No = 0; Yes =1 | Dichotomous |
| Does your school or any other organisation provide any extracurricular physical activity or sports programmes available to Year 9 during lunch breaks? | No = 0; Yes =1 | Dichotomous |
| Does your school or any other organisation provide any extracurricular physical activity or sports programmes available to Year 9 after school? | No = 0; Yes =1 | Dichotomous |
| Does your school or any other organisation provide any extracurricular physical activity or sports programmes available to Year 9 at weekends? | No = 0; Yes =1 | Dichotomous |
| Availability of rounders as extracurricular activity? | No = 0; Yes =1 | Dichotomous |
| Availability of cricket as extracurricular activity? | No = 0; Yes =1 | Dichotomous |
| Availability of table tennis as extracurricular activity? | No = 0; Yes =1 | Dichotomous |
| Availability of gymnastics as extracurricular activity? | No = 0; Yes =1 | Dichotomous |
| Availability of boxing as extracurricular activity? | No = 0; Yes =1 | Dichotomous |
| Availability of volleyball as extracurricular activity? | No = 0; Yes =1 | Dichotomous |
| Availability of swimming as extracurricular activity? | No = 0; Yes =1 | Dichotomous |
| Availability of archery as extracurricular activity? | No = 0; Yes =1 | Dichotomous |
| Availability of martial arts as extracurricular activity? | No = 0; Yes =1 | Dichotomous |
| Availability of dodgeball as extracurricular activity? | No = 0; Yes =1 | Dichotomous |
| Availability of fencing as extracurricular activity? | No = 0; Yes =1 | Dichotomous |
| Availability of handball as extracurricular activity? | No = 0; Yes =1 | Dichotomous |
| Availability of Ultimate Frisbee as extracurricular activity? | No = 0; Yes =1 | Dichotomous |
| Availability of yoga as extracurricular activity? | No = 0; Yes =1 | Dichotomous |
| Availability of Zumba as extracurricular activity? | No = 0; Yes =1 | Dichotomous |
| Availability of Pilates as extracurricular activity? | No = 0; Yes =1 | Dichotomous |
| Availability of badminton as extracurricular activity? | No = 0; Yes =1 | Dichotomous |
| Availability of dance as extracurricular activity? | No = 0; Yes =1 | Dichotomous |
| Availability of running as extracurricular activity? | No = 0; Yes =1 | Dichotomous |
| Availability of trampolining as extracurricular activity | No = 0; Yes =1 | Dichotomous |
| Availability of tennis as extracurricular activity? | No = 0; Yes =1 | Dichotomous |
| Availability of hockey as extracurricular activity? | No = 0; Yes =1 | Dichotomous |
| Availability of football as extracurricular activity? | No = 0; Yes =1 | Dichotomous |
| Availability of netball as extracurricular activity? | No = 0; Yes =1 | Dichotomous |
| Availability of rugby as extracurricular activity? | No = 0; Yes =1 | Dichotomous |
| Availability of athletics as extracurricular activity? | No = 0; Yes =1 | Dichotomous |
| Availability of other sports as extracurricular activities? | No = 0; Yes =1 | Dichotomous |
| Availability of basketball as extracurricular activity? | No = 0; Yes =1 | Dichotomous |
| Availability of cheerleading as extracurricular activities? | No = 0; Yes =1 | Dichotomous |
| My school considers it important to encourage pupils to be physically active at school (for example, during school breaks)? | Strongly disagree = 1; Disagree = 2; Neither agree nor disagree = 3; Agree = 4; Strongly agree = 5 | Categorical |
| My school considers it important to encourage pupils to do physical activity outside of school | Strongly disagree = 1; Disagree = 2; Neither agree nor disagree = 3; Agree = 4; Strongly agree = 5 | Categorical |
| My school considers it important to educate pupils about the risks of inactivity | Strongly disagree = 1; Disagree = 2; Neither agree nor disagree = 3; Agree = 4; Strongly agree = 5 | Categorical |
| My school considers it important to provide information on how to be physically active in a safe manner | Strongly disagree = 1; Disagree = 2; Neither agree nor disagree = 3; Agree = 4; Strongly agree = 5 | Categorical |
| My school considers it important to encourage pupils to use active transport to school (e.g. walking, cycling) | Strongly disagree = 1; Disagree = 2; Neither agree nor disagree = 3; Agree = 4; Strongly agree = 5 | Categorical |
| Which of the following statements best describes your rules relating to where Year 9 pupils can go during breaks (including lunchtime)? | It is compulsory for all Year 9 pupils to go outside, irrespective of the weather = 1; When the weather allows, it is compulsory for all Year 9 pupils to go outside. However, all Year 9 pupils are kept inside in bad weather = 2; When the weather allows, it is compulsory for all Year 9 pupils to go outside. However, if the weather is bad, they are allowed inside or outside = 3; The Year 9 pupils are allowed to go both inside and outside, irrespective of the weather = 4; It is compulsory for all Year 9 pupils to stay inside, irrespective of the weather = 5 | Categorical |
| Are the Year 9 pupils allowed to use a computer during breaks? | Yes, always = 1; Yes, in bad weather = 2; No, never = 3; | Categorical |
| Are the Year 9 pupils allowed to watch TV or videos during breaks? | Yes, always = 1; Yes, in bad weather = 2; No, never = 3; | Categorical |
| Are the Year 9 pupils allowed to use the school’s sports equipment during breaks? | Yes, always = 1; Yes, in bad weather = 2; No, never = 3; | Categorical |
| Are the Year 9 pupils allowed to play ball games indoors during breaks? | Yes, always = 1; Yes, in bad weather = 2; No, never = 3; | Categorical |
| Are the Year 9 pupils allowed to play ball games outdoors during breaks? | Yes, always = 1; Yes, in bad weather = 2; No, never = 3; | Categorical |
| Does your school have a policy to promote PA among Year 9 pupils? | Yes, a written policy = 1; Yes, an informal policy = 2; No =3 | Categorical |
| During a normal week how often do the following things happen? My friends do physical activity or play sports with me. | 1 = Never or hardly ever; 2 = Once or twice a week; 3 = Nearly every day; 4 = Every day; | Categorical |
| During a normal week how often do the following things happen? I ask my friends to do physical activities or play sports with me. | 1 = Never or hardly ever; 2 = Once or twice a week; 3 = Nearly every day; 4 = Every day; | Categorical |
| During a normal week how often do the following things happen? My friends ask me to do physical activities or play sports with them. | 1 = Never or hardly ever; 2 = Once or twice a week; 3 = Nearly every day; 4 = Every day; | Categorical |
| Number of pupils 2016-2017 | Free text e.g. 1000 pupils = 1000 | Continuous |
| Number of pupils 2016-2017 | Free text e.g. 100 teachers = 100 | Continuous |
| School total expenditure 2016-2017 (£) | Free text e.g. £6.2m = 6200000 | Continuous |
| Staff total expenditure 2016-2017 (£) | Free text e.g. £4.38m = 4380000 | Continuous |
| Premises total expenditure 2016-2017 (£) | Free text e.g. £415k = 415000 | Continuous |
| Occupation total expenditure 2016-2017 (£) e.g. the costs associated with occupying the school building (energy, water, sewerage, rates, insurance, and catering) | Free text e.g. £616k = 616000 | Continuous |
| Supplies and services total expenditure 2016-2017 (£) | Free text e.g. £950k = 950000 | Continuous |
| Cost of finance expenditure 2016-2017 (£) | Free text e.g. £175k = 175000 | Continuous |
| Special facilities expenditure 2016-2017 (£) | Free text e.g. £3.75k = 3750 | Continuous |
| Teaching staff expenditure 2016-2017 (£) | Free text e.g. £3.23m = 3230000 | Continuous |
| Supply staff expenditure 2016-2017 (£) | Free text e.g. £184k = 184000 | Continuous |
| Education support staff expenditure 2016-2017 (£) | Free text e.g. £744k = 744000 | Continuous |
| Administrative and clerical staff expenditure 2016-2017 (£) | Free text e.g. £746k = 746000 | Continuous |
| Other staff costs expenditure 2016-2017 (£) e.g. this includes cost of other staff, indirect employee expenses, staff development and training) | Free text e.g. £283k = 283000 | Continuous |
| Premises staff expenditure 2016-2017 (£) | Free text e.g, £189k = 189000 | Continuous |
| Cleaning and caretaking staff expenditure 2016-2017 (£) | Free text e.g. 106k = 106000 | Continuous |
| Maintenance and improvement expenditure 2016-2017 (£) | Free text e.g. 146k = 146000 | Continuous |
| PFI charges 2016-2017 (£) | Free text e.g. £0 = 0 | Continuous |
| Energy expenditure 2016-2017 (£) e.g. all costs related to fuel and energy | Free text e.g. £107k = 107000 | Continuous |
| Water and sewerage expenditure 2016-2017 (£) | Free text e.g. £17k = 17000 | Continuous |
| Other occupation costs expenditure 2016-2017 (£) e.g. rents for premises, refuse collection, hygiene services) | Free text e.g. $16k = 16000 | Continuous |
| Other insurance premiums expenditure 2016-2017 (£) e.g. premises related insurance, vehicle insurance, school trip insurance) | Free text e.g. £36k = 36000 | Continuous |
| Catering expenditure 2016-2017 (£) | Free text e.g. £163k = 163000 | Continuous |
| Rents and rates expenditure 2016-2017 (3) e.g. business rates, national non-domestic rates) | Free text e.g. £50k = 50000 | Continuous |
| Administrative supplies expenditure 2016-2017 (£) | Free text e.g. £255k = 255000 | Continuous |
| Educational supplies expenditure 2016-2017 (£) | Free text e.g. £474k = 474000 | Continuous |
| Bought in professional services expenditure 2016-2017 (£) e.g. educational consultancy, auditor costs) | Free text e.g. £116k = 116000 | Continuous |
| Total income 2016-2017 (£) | Free text e.g. £6m = 6000000 | Continuous |
| Grant funding total 2016-2017 (£) | Free text e.g. £5m 5000000 | Continuous |
| Self-generated funding total 2016-2017 (£) | Free text e.g. £349k = 340000 | Continuous |
| In year balance 2016-2017 (£) | Free text e.g. £220k = 220000 | Continuous |
| Revenue reserve 2016-2017 (£) | Free text e.g. £444k = 444000 | Continuous |
| Direct grants 2016-2017 (£) e.g. DfE/EFA revenue grants, pre-16 funding | Free text e.g. £5.3m = 5300000 | Continuous |
| Community grants 2016-2017 (£) | Free text e.g. £370k = 370000 | Continuous |
| Targeted grants 2016-2017 (£) | Free text e.g. £240k = 240000 | Continuous |
| Income from facilities and services 2016-2017 (£) e.g. income from meals provided to external customers, income for consultancy, training courses and examination fees | Free text e.g. £150k = 150000 | Continuous |
| Income from catering 2016-2017 (£) | Free text e.g. £220k = 220000 | Continuous |
| Donations and/or voluntary funds 2016-2017 (£) | Free text e.g. $£100k = 100000 | Continuous |
| Receipts from supply teacher insurance claims 2016-2017 (3) | Free text e.g. £1k = 1000 | Continuous |
| Investment income 2016-2017 (£) | Free text e.g. £9k = 9000 | Continuous |
| Other self-generated income 2016-2017 (£) | Free text e.g. £57k = 57000 | Continuous |
| School workforce full time equivalent 2016-2017 (Full time equivalent) | Free text e.g. 101FTE = 101 | Continuous |
| Total number of teachers 2016-2017 (full time equivalent) | Free text e.g. 62FTE = 62 | Continuous |
| Teachers with qualified teacher status 2016-2017 (5) | Free text e.g. 99% = 99 | Continuous |
| Senior leadership 2016-2017 (full time equivalent) | Free text e.g. 8FTE = 8 | Continuous |
| Teaching assistants 2016-2017 (full time equivalent) | Free text e.g. 11FTE = 11 | Continuous |
| Non-classroom support staff excluding auxiliary staff 2016-2017 (full time equivalent) | Free text e.g. 31TE = 31 | Continuous |
| Auxiliary staff 2016-2017 (full time equivalent) | Free text e.g. 13FTE = 13 | Continuous |
| School work force head count 2016-2017 | Free text e.g. 148 | Continuous |
| OFSTED Rating | 1 = Outstanding; 2 = Good; 3 = Requires Improvement; 4 = Inadequate | Categorical |
| Number of pupils 2016-2017 | Free text e.g. 1000 pupils = 1000 | Continuous |
| Number of teachers 2016-2017 | Free text e.g. 100 teachers = 100 | Continuous |
| School total expenditure 2017-2018 (£) | Free text e.g. £6.2m = 6200000 | Continuous |
| Staff total expenditure 2017-2018 (£) | Free text e.g. £4.38m = 4380000 | Continuous |
| Premises total expenditure 2017-2018 (£) | Free text e.g. £415k = 415000 | Continuous |
| Occupation total expenditure 2017-2018 (£) e.g. the costs associated with occupying the school building (energy, water, sewerage, rates, insurance, and catering) | Free text e.g. £616k = 616000 | Continuous |
| Supplies and services total expenditure 2017-2018 (£) | Free text e.g. £950k = 950000 | Continuous |
| Cost of finance expenditure 2017-2018 (£) | Free text e.g. £175k = 175000 | Continuous |
| Special facilities expenditure 2017-2018 (£) | Free text e.g. £3.75k = 3750 | Continuous |
| Teaching staff expenditure 2017-2018 (£) | Free text e.g. £3.23m = 3230000 | Continuous |
| Supply staff expenditure 2017-2018 (£) | Free text e.g. £184k = 184000 | Continuous |
| Education support staff expenditure 2017-2018 (£) | Free text e.g. £744k = 744000 | Continuous |
| Administrative and clerical staff expenditure 2017-2018 (£) | Free text e.g. £746k = 746000 | Continuous |
| Other staff costs expenditure 2017-2018 (£) e.g. this includes cost of other staff, indirect employee expenses, staff development and training) | Free text e.g. £283k = 283000 | Continuous |
| Premises staff expenditure 2017-2018 (£) | Free text e.g, £189k = 189000 | Continuous |
| Cleaning and caretaking staff expenditure 2017-2018 (£) | Free text e.g. 106k = 106000 | Continuous |
| Maintenance and improvement expenditure 2017-2018 (£) | Free text e.g. 146k = 146000 | Continuous |
| PFI charges 2017-2018 (£) | Free text e.g. £0 = 0 | Continuous |
| Energy expenditure 2017-2018 (£) e.g. all costs related to fuel and energy | Free text e.g. £107k = 107000 | Continuous |
| Water and sewerage 2017-2018 (£) | Free text e.g. £17k = 17000 | Continuous |
| Other occupation costs expenditure 2017-2018 (£) e.g. rents for premises, refuse collection, hygiene services) | Free text e.g. $16k = 16000 | Continuous |
| Other insurance premiums expenditure 2017-2018 (£) e.g. premises related insurance, vehicle insurance, school trip insurance) | Free text e.g. £36k = 36000 | Continuous |
| Catering expenditure 2017-2018 (£) | Free text e.g. £163k = 163000 | Continuous |
| Rents and rates expenditure 2017-20178 (3) e.g. business rates, national non-domestic rates | Free text e.g. £50k = 50000 | Continuous |
| Administrative supplies expenditure 2017-2018 (£) | Free text e.g. £255k = 255000 | Continuous |
| Educational supplies expenditure 2017-2018 (£) | Free text e.g. £474k = 474000 | Continuous |
| Bought in professional services expenditure 2017-2018 (£) e.g. educational consultancy, auditor costs) | Free text e.g. £116k = 116000 | Continuous |
| Total income 2017-2018 (£) | Free text e.g. £6m = 6000000 | Continuous |
| Grant funding total 2017-2018 (£) | Free text e.g. £5m 5000000 | Continuous |
| Self-generated funding total 2017-2018 (£) | Free text e.g. £349k = 340000 | Continuous |
| In year balance 2017-2018 (£) | Free text e.g. £220k = 220000 | Continuous |
| Revenue reserve 2017-2018 (£) | Free text e.g. £444k = 444000 | Continuous |
| Direct grants 2017-2018 (£) e.g. DfE/EFA revenue grants, pre-16 funding | Free text e.g. £5.3m = 5300000 | Continuous |
| Community grants 2017-2018 (£) | Free text e.g. £370k = 370000 | Continuous |
| Targeted grants 2017-2018 (£) | Free text e.g. £240k = 240000 | Continuous |
| Income from facilities and services 2017-2018 (£) e.g. income from meals provided to external customers, income for consultancy, training courses and examination fees | Free text e.g. £150k = 150000 | Continuous |
| Income from catering 2017-2018 (£) | Free text e.g. £220k = 220000 | Continuous |
| Donations and of voluntary funds 2017-2018 (£) | Free text e.g. $£100k = 100000 | Continuous |
| Receipts from supply teacher insurance claims 2017-2018 (3) | Free text e.g. £1k = 1000 | Continuous |
| Investment income 2017-2018 (£) | Free text e.g. £9k = 9000 | Continuous |
| Other self-generated income 2017-2018 (£) | Free text e.g. £57k = 57000 | Continuous |
| School workforce full time equivalent 2017-2018 (Full time equivalent) | Free text e.g. 101FTE = 101 | Continuous |
| Total number of teachers 2017-2018 (full time equivalent) | Free text e.g. 62FTE = 62 | Continuous |
| Teachers with qualified teacher status 2017-2018 (5) | Free text e.g. 99% = 99 | Continuous |
| Senior leadership 2017-2018 (full time equivalent) | Free text e.g. 8FTE = 8 | Continuous |
| Teaching assistants 2017-2018 (full time equivalent) | Free text e.g. 11FTE = 11 | Continuous |
| Non-classroom support staff excluding auxiliary staff 2017-2018 (full time equivalent) | Free text e.g. 31TE = 31 | Continuous |
| Auxiliary staff 2017-2018 (full time equivalent) | Free text e.g. 13FTE = 13 | Continuous |
| School work force head count 2017-2018 | Free text e.g. 148 | Continuous |
| Change in number of pupils between 2016-2017 and 2017-2018 | Free text e.g. -50 pupils = -50 | Continuous |
| Change in number of teachers between 2016-2017 and 2017/2018 | Free text e.g. 5 teachers = 5 | Continuous |
| Change in school total expenditure between 2016-2017 and 2017-2018 (£) | Free text e.g. £-200k = -200000 | Continuous |
| Change in staff total expenditure between 2016-2017 and 2017-2018 (£) | Free text e.g. £-14k = -14000 | Continuous |
| Change in premises total expenditure between 2016-2017 and 2017-2018 (£) | Free text e.g. £11k = 11000 | Continuous |
| Change in occupation total expenditure between 2016-2017 and 2017-2018 (£) e.g. change in the costs associated with occupying the school building (energy, water, sewerage, rates, insurance, and catering) | Free text e.g. £10k = 10000 | Continuous |
| Change in supplies and services total expenditure between 2016-2017 and 2017-2018 (£) | Free text e.g. £65k = 65000 | Continuous |
| Change in cost of finance expenditure 2016-2017 and 2017-2018 (£) | Free text e.g. £19k = 19000 | Continuous |
| Change in special facilities expenditure 2016-2017 and 2017-2018 (£) | Free text e.g. £-9k -8000 | Continuous |
| Change in teaching staff expenditure between 2016-2017 and 2017-2018 (£) | Free text e.g. £-26K = -26000 | Continuous |
| Change in supply staff expenditure between 2016-2017 and 2017-2018 (£) | Free text e.g. £-10K = -10000 | Continuous |
| Change in education support staff expenditure between 2016-2017 and 2017-2018 (£) | Free text e.g. £-41k = 41000 | Continuous |
| Change in administrative and clerical staff expenditure between 2016-2017 and 2017-2018 (£) | Free text e.g. £-28K = -28000 | Continuous |
| Change in other staff costs expenditure between 2016-2017 and 2017-2018 (£) e.g. change in the cost of other staff, indirect employee expenses, staff development and training) | Free text e.g. £-148K = -148000 | Continuous |
| Change in premises staff expenditure between 2016-2017 and 2017-2018 (£) | Free text e.g. £-61K = -61000 | Continuous |
| Change in cleaning and caretaking staff expenditure between 2016-2017 and 2017-2018 (£) | Free text e.g. £1k = 1000 | Continuous |
| Change in maintenance and improvement expenditure between 2016-2017 and 2017-2018 (£) | Free text e.g. £5k = 5000 | Continuous |
| Change in PFI charges between 2016-2017 and 2017-2018 (£) | Free text e.g. £59K = 59000 | Continuous |
| Change in energy expenditure between 2016-2017 and 2017-2018 (£) e.g. all costs related to fuel and energy | Free text e.g. £40K = 40000 | Continuous |
| Change in water and sewerage between 2016-2017 and 2017-2018 (£) | Free text e.g. £4k = 4000 | Continuous |
| Change in other occupation costs expenditure between 2016-2017 and 2017-2018 (£) e.g. rents for premises, refuse collection, hygiene services) | Free text e.g. £3k = 3000 | Continuous |
| Change in other insurance premiums expenditure between 2016-2017 and 2017-2018 (£) e.g. change in premises related insurance, vehicle insurance, school trip insurance) | Free text e.g. £7k = 7000 | Continuous |
| Change in catering expenditure between 2016-2017 and 2017-2018 (£) | Free text e.g. £36k = 36000 | Continuous |
| Change in rents and rates expenditure between 2016-2017 and 2017-2018 (3) e.g. change in business rates, national non-domestic rates) | Free text e.g. £4k = 4000 | Continuous |
| Change in administrative supplies expenditure between 2016-2017 and 2017-2018 (£) | Free text e.g. £77k = 77000 | Continuous |
| Change in educational supplies expenditure between 2016-2017 and 2017-2018 (£) | Free text e.g. £-47k = -47000 | Continuous |
| Change in bought in professional services expenditure between 2016- 2017 and 2017-2018 (£) e.g. educational consultancy, auditor costs) | Free text e.g. £750 = 750 | Continuous |
| Change in total income between 2016-2017 and 2017-2018 (£) | Free text e.g. £85k = 85000 | Continuous |
| Change in grant funding total between 2016-2017 and 2017-2018 (£) | Free text e.g. £111k = 111000 | Continuous |
| Change in self-generated funding total between 2016-2017 and 2017-2018 (£) | Free text e.g. £-10k = 10000 | Continuous |
| Change in in year balance between 2016-2017 and 2017-2018 (£) | Free text eg. £100k = 100000 | Continuous |
| Change in revenue reserve between 2016-2017 and 2017-2018 (£) | Free text e.g. £-21k = -21000 | Continuous |
| Change in direct grants between 2016-2017 and 2017-2018 (£) e.g. change in DfE/EFA revenue grants, pre-16 funding | Free text e.g. £88k = 88000 | Continuous |
| Change in community grants between 2016-2017 and 2017-2018 (£) | Free text e.g. £-20k = 20000 | Continuous |
| Change in targeted grants between 2016-2017 and 2017-2018 (£) | Free text e.g. £6k = 6000 | Continuous |
| Change in income from facilities and services between 2016-2017 and2017-2018 (£) e.g. change in income from meals provided to external customers, income for consultancy, training courses and examination fees | Free text e.g. £5k = 5000 | Continuous |
| Change in income from catering between 2016-2017 and 2017-2018 (£) | Free text e.g. £32k = 32000 | Continuous |
| Change in donations and/or voluntary funds between 2016-2017 and 2017-2018 (£) | Free text e.g. £-10k = 10000 | Continuous |
| Change in receipts from supply teacher insurance claims between 2016-2017 and 2017-2018 (3) | Free text e.g. 0 | Continuous |
| Change in investment income between 2016-2017 and 2017-2018 (£) | Free text e.g. £400 = 400 | Continuous |
| Change in other self-generated income between 2016-2017 and 2017-2018 (£) | Free text e.g. £-47k = 47000 | Continuous |
| Change in school workforce full time equivalent between 2016-2017 and 2017-2018 (Full time equivalent) | Free text e.g. 18.7FTE = 18.7 | Continuous |
| Change in total number of teachers between 2016-2017 and 2017-2018 (full time equivalent) | Free text e.g. 4.4. | Continuous |
| Change in teachers with qualified teacher status between 2016-2017 and 2017-2018 (%) | Free text e.g. 3.3 | Continuous |
| Change in senior leadership between 2016-2017 and 2017-2018 (full time equivalent) | Free text e.g. -1 | Continuous |
| Change in teaching assistants between 2016-2017 and 2017-2018 (full time equivalent) | Free text e.g. -1.4 | Continuous |
| Change in non-classroom support staff excluding auxiliary staff between 2016-2017 and 2017-2018 (full time equivalent) | Free text e.g. 1 | Continuous |
| Change in auxiliary staff between 2016-2017 and 2017-2018 (full time equivalent) | Free text e.g. 2 | Continuous |
| Change in school work force head count between 2016-2017 and 2017-2018 | Free text e.g. 10 | Continuous |
