# Supplementary material for: The school policy, social, and physical environment and change in adolescent physical activity: An exploratory analysis using the LASSO
Source: PLoS One. 2021 Apr 8;16(4):e0249328. doi: 10.1371/journal.pone.0249328 (PMC8031174; doi:10.1371/journal.pone.0249328)
Supplement: S1 File — (PDF) [file pone.0249328.s002.pdf]

Version 1 10.05.2016

ID

Thank you for agreeing to participate in the GoActive evaluation study.

In this booklet, we will be asking short questions about the physical activity opportunities in your school.

- ✓ Please complete all of the questions in this booklet.
- ✓ Please only select one answer per question or item (i.e. either tick *one* box, or circle *one* response).

Please consider Year 9 in particular when answering this questionnaire.

Your answers will be treated as confidential. If you have any questions, please do not hesitate to contact the study team: goactive@mrc-epid.cam.ac.uk
or Freephone 0800 917 3319

NHS National Institute for Health Research
UNIVERSITY OF CAMBRIDGE
MRC Epidemiology Unit
CEDAR Centre for Diet and Activity Research
A UKCRC Public Health Research Centre of Excellence

Section 1: School information

1. Name of school ________________________________

2. What is your position?

- ☐ Head teacher
- ☐ Deputy head teacher
- ☐ Physical Education lead
- ☐ Year 9 lead
- ☐ Other (please specify)____

3. What time does the normal school day start? __________ and finish? __________

4. At what time are breaks held, and how long do they last?

	Start time	Duration (minutes)
a. Morning break		
b. Lunchtime		
c. Afternoon break		
d. Other (please specify) ___________________		

5. Have any events occurred during the measurement period that may have influenced the level of physical activity of Year 9 students (e.g. sports day)?

Your measurement period: ________________ to ________________

- ☐ No
- ☐ Yes (*please give details*)

....

....

....

....

Please now think about the area around your school.

6. Please indicate whether the following are present:

None	Some	A lot	
			a. Planted beds containing flowers/shrubs/small trees
			b. Trees for shade
			c. Loud ambient noise (e.g. traffic, trains, industry)
			d. Litter
			e. Murals/outdoor art
			f. Graffiti

7. To what extent do you agree or disagree with the following statements?

	Strongly disagree	Disagree	Neither disagree nor agree	Agree	Strongly agree
a. The grounds are shielded from the surrounding area by hedges/trees/fences	☐	☐	☐	☐	☐
b. The grounds are generally well maintained	☐	☐	☐	☐	☐
c. The grounds are generally free of vandalism	☐	☐	☐	☐	☐

Section 2: Pupil overview

Please answer the following questions in reference to Year 9 wherever possible.

If Year 9 information is not available, please complete school-level information.

8. How many pupils are there in….?	a. Year 9…………	b. Whole school…………
9. How many boys are there in…?	a. Year 9…………	b. Whole school…………
10. How many girls are there in…?	a. Year 9…………	b. Whole school…………
11. What is the percentage of pupils for whom you receive Pupil Premium funding?	a. Year 9…………	b. Whole school…………

Section 3: Physical activity opportunities at school

12. Does your school have access to…

Please tick all that apply.

If yes, how would you rate their quality?

Please take into account the level of maintenance, cleanliness, accessibility etc.

	No	Yes		High	Medium	Low
a. A specific indoor hall for gym or sports			→			
b. A shared indoor facility used for sports activities			→			
c. A sports or football field/pitch on school grounds			→			
d. Athletics track (grass or hard surface)			→			
e. Courts (e.g. tennis, basketball including half court, netball, multicourt area)			→			
f. A recreational area on school grounds			→			
g. A wildlife garden			→			
h. Bright or fluorescent markings on play surfaces (e.g. hopscotch, animals)			→			
i. Playground equipment (e.g. swings, slide)			→			
j. Benches			→			
k. Picnic tables			→			
l. Drinking fountains			→			
m. Uncovered cycle parking			→			
n. Covered cycle parking			→			
o. An assault course			→			
p. Formal garden/quiet space			→			
q. Outdoor teaching space			→			
r. Vegetable/fruit garden			→			
s. Playing fields or a local park off school grounds, which you can use			→			
t. Purpose built changing facilities			→			
u. Sports equipment (e.g. gymnastics equipment)			→			

13. Are the school grounds generally suitable for….?

Very	Somewhat	Not at all	
			a. Sport (organised or not)
			b. Informal games (kickabout, Frisbee etc.)
			c. General play

14. How many hours of physical education do the pupils in Year 9 usually have per week?

Please round to the nearest half hour.

…………………………… hours per week

15. Does your school or any other organisation provide any extracurricular physical activity or sports programmes available to Year 9?

Please tick your response in each case.

	No	Yes
a. Before school		
b. During lunch breaks		
c. After school		
d. At weekends		

16. Which of the following physical activities or sports are available as extracurricular programmes?

Please tick any that apply.

- ☐ a. Rounders
- ☐ b. Cricket
- ☐ c. Table tennis
- ☐ d. Gymnastics
- ☐ e. Boxing
- ☐ f. Volleyball
- ☐ g. Swimming
- ☐ h. Archery
- ☐ i. Martial Arts

  Please specify

  ………………………………

- ☐ j. Dodgeball
- ☐ k. Fencing
- ☐ l. Handball
- ☐ m. Ultimate Frisbee
- ☐ n. Yoga
- ☐ o. Zumba
- ☐ p. Pilates
- ☐ q. Badminton
- ☐ r. Dance

  Please specify

  ………………………………

- ☐ s. Tennis
- ☐ t. Hockey
- ☐ u. Football
- ☐ v. Netball
- ☐ w. Rugby
- ☐ x. Athletics
- ☐ y. Other

  Please specify

  ………………………………

Section 4: School rules and attitudes

Please tick the box that best indicates your agreement or disagreement with each of the following statements.

17. My school considers it important to…

	Strongly disagree	Disagree	Neither agree nor disagree	Agree	Strongly agree
a…encourage pupils to be physically active at school (for example, during school breaks)					
b…encourage pupils to do physical activity outside of school					
c…educate pupils about the risks of physical inactivity					
d…provide information on how to be physically active in a safe manner					
e…encourage pupils to use active transport to school (e.g. walking, cycling)					

18. Which of the following statements best describes your rules relating to where Year 9 pupils can go during breaks (including lunchtime)?

Please tick one box only.

	It is compulsory for all Year 9 pupils to go outside, irrespective of the weather.
	When the weather allows, it is compulsory for all Year 9 pupils to go outside. However, all Year 9 pupils are kept inside in bad weather.
	When the weather allows, it is compulsory for all Year 9 pupils to go outside. However, if the weather is bad, they are allowed inside or outside.
	The Year 9 pupils are allowed to go both inside and outside, irrespective of the weather.
	It is compulsory for all Year 9 pupils to stay inside, irrespective of the weather.

19. Are the Year 9 pupils allowed to do the following during breaks?

Please tick only one box per statement.

	Yes, always	Yes, in bad weather	No, never
a. Use a computer	☐	☐	☐
b. Watch TV or videos	☐	☐	☐
c. Use the school's sports equipment	☐	☐	☐
d. Play ball games indoors	☐	☐	☐
e. Play ball games outdoors	☐	☐	☐

20. Does your school have a policy to promote physical activity among Year 9 pupils?

Please tick only one box.

☐	Yes, a written policy
☐	Yes, an informal policy
☐	No

Thank you very much for completing this questionnaire – we really appreciate your time!
